# The Impact of Rice Lipid on In Vitro Rice Starch Digestibility

**DOI:** 10.3390/foods11101528

**Published:** 2022-05-23

**Authors:** Amina Khatun, Daniel L. E. Waters, Lei Liu

**Affiliations:** 1 Southern Cross Plant Science, Faculty of Science and Engineering, Southern Cross University, Lismore, NSW 2480, Australia; amina.khatun@scu.edu.au (A.K.); daniel.waters@scu.edu.au (D.L.E.W.); 2 Southern Cross Analytical Research Services, Southern Cross University, Lismore, NSW 2480, Australia

**Keywords:** diacylglycerol, lysophospholipid, phospholipid, starch–lipid complex, starch digestion, triacylglycerol

## Abstract

The negative role of lipids in rice starch digestion is well-known; however, the effect of individual native lipids on starch digestibility has not been studied. In this study, native rice lipids, such as triacylglycerols (TAGs), diacylglycerols (DAGs), phosphatidylcholines (PCs) and lysophospholipids (LPLs), were analyzed using liquid chromatography–mass spectrometry (LC-MS) and correlated with in vitro rice starch digestibility. Most of the tested lipids exhibited a negative correlation with the in vitro starch digestibility with the correlations being more pronounced for LPLs. Removal of lipids from rice flour increased the in vitro starch digestibility. Conversely, a lipid extract addition to rice flour reduced the starch digestibility. Addition of 1% pure lysophosphatidylcholine (LPC)16:0, TAG54:6, DAG36:4 or PC36:2 individually to rice flour reduced starch digestibility by different extents in the order of LPC16:0 > TAG54:6 > PC36:2 > DAG36:4. LPC16:0 was the most abundant lipid among all the assessed lipids in the white rice (milled rice), and addition of 1% LPC 16:0 to rice flour reduced glucose release following three hours of in vitro starch digestion by 7.4%. There may be a scope to breed rice with a lipid composition to reach a desired starch digestibility or simply through addition of certain lipids before cooking the rice.

## 1. Introduction

Rice starch is a major source of dietary calories, and rice starch digestibility has a great impact on global health and nutrition [1]. Rice starch digestibility can be dependent upon starch granule size, crystallinity, amylose content and the chain length of amylopectin [2,3,4]. Rice grain also contains non-starch components such as proteins, lipids and fibre [5]. Since most of the commercial rice varieties contain a similar starch content, the wide range of rice starch digestibility found in these rices may be due to non-starch components [6].

Lipids are the second major non-starch component in rice grain after protein and are more concentrated in brown rice (~3% of dry weight) than milled rice (~1% of dry weight) [7,8]. Rice lipids are mostly non-polar; however, some lipids such as phospholipids and glycolipids contain a polar moiety [7]. These polar lipids may have different effects on rice starch digestibility.

Addition of non-rice lipids to rice decreases starch digestibility [9]. Traditionally, lipids such as soybean oil and clarified butter (ghee) are added to rice, which may affect starch digestibility [10]. Addition of these oils to brown and milled rice before, during or after cooking slowed the in vitro starch digestion rate, with the addition of ghee before or during boiling of brown rice showing the lowest level of glucose release [10]. Addition of palm oil to brown, black, milled and waxy rice decreased rapidly digestible starch (RDS) and slowly digestible starch (SDS) and increased the resistant starch (RS) content, except in waxy rice [10,11]. Chen et al. added maize oil to rice flour and starch cooked at 20%, 30% and 40% moisture content [12]. Addition of lipids decreased RDS and increased SDS and RS in cooked rice starch and flour, with the highest levels measured at 20% moisture content [12]. The impact of adding non-rice lipids to rice starch digestion may depend on the time at which the lipids were added, the type of rice starch (waxy and non-waxy) and the amount of moisture during cooking. However, since the lipids added to rice in these studies were different from native rice grain lipids, their influence on rice starch digestibility may differ.

Lipids may decrease starch digestibility by the formation of a lipid–starch complex [13,14], which would depend on the polarity of lipids. Difficulties in extraction of lipids in ambient conditions suggest some lipids may form a complex with starch molecules inside rice starch granules [15]. Some non-polar lipids present outside starch granules called non-starch lipids are easily extracted with organic solvent at room temperature [16,17]. However, some polar lipids, mainly free fatty acids and lysophospholipids (LPLs), are present inside starch granules, called starch lipids [17], and need to be extracted with water and heat [17,18]. Some non-starch lipids, mainly phospholipids, glycolipids and triacylglycerols (TAGs), are on the surface of starch granules, and these lipids are sometimes called starch surface lipids [16,19].

Although lipids are known to modulate starch digestibility, the effects of individual lipid classes native to the rice grain on rice starch digestibility have not been compared. Ethanol, a polar organic solvent, can remove starch granule surface lipids such as phospholipids, and Hu et al. removed lipids from rice starch with ethanol and found increased in vitro starch digestibility [20]. Petroleum ether, a non-polar organic solvent, only removes non-starch lipids, and Ye et al. removed lipids from rice flour with petroleum ether and found the remaining rice flour had a higher in vitro starch digestibility than the untreated flour [21]. It is difficult to compare the effects of rice phospholipids and TAGs on rice starch digestibility from these two studies as these solvents can extract other compounds which may have effects on starch digestibility. In the research reported here, rice lipids such as TAGs, diacylglycerol (DAG), phospholipids and LPLs were extracted from rice flour separately using different organic solvents and quantified using liquid chromatography–mass spectrometry (LC-MS), and their amounts were then correlated with in vitro starch digestibility. Confirmation of their effects on rice starch digestibility was achieved by addition of lipid extracts to rice flour and addition of pure standards of lipids found in rice flour.

## 2. Materials and Methods

### 2.1. Materials, Solvents, Reagents and Standards

In total, 25 milled rice (also known as polished rice or white rice) samples were used in this study (Table 1). The rice samples included commercially purchased rice from Australian market (Table 1, R01–R10) and rice samples grown in different parts of Bangladesh (Table 1, R11–R25). The purchased rice from Australian market (Table 1, R01–R10) comprised major rice varieties which were commercially grown, processed, packed and marketed, reflecting generally consumed rice in Australia. The rice obtained from Bangladesh (Table 1, R11–R25) comprised 14 polished rices provided by the Bangladesh Rice Research Institute (BRRI) and 1 rice sample provided by Purnava Limited, Bangladesh (dehulled by mortar and pestle and polished using a rice-milling machine, model: JNNJ3B, LZHZXY, Taizhou, China). The commercial Jasmine long-grain rice (Table 1, R04) was used to develop the method for rice lipid extraction and the method for lipid analysis by liquid chromatography–mass spectrometry (LC-MS). The commercial Doongara rice (Table 1, R02) was a low-glycaemic-index (GI) rice bred in Australia. The Doongara rice was selected in the study to evaluate the effect of adding or removing lipids on in vitro rice starch digestibility and to provide the information on whether changing lipids in rice could further lower the GI.

All organic solvents were of high-performance-liquid-chromatography (HPLC) grade. Ammonium formate was purchased from Aldrich Chemical Company Inc. Trichloroacetic acid was purchased from Merck, Darmstadt, Germany, and trifluoroacetic from Sigma-Aldrich Co., St. Louis, MO, USA. Lipid standards (Table 2), triacylglycerols (TAG 54:3 and TAG 54:6), diacylglycerol (DAG 36:4) and phosphatidylcholines (PC 36:2 and PC 34:1) and rat intestinal acetone powder (I1630) were purchased from Sigma-Aldrich Co., St. Louis, MO, USA. Standard lysophospholipids (lysophosphatidylcholine, LPC: 16:0 and LPC 18:1; lysophosphatidylethanolamine, LPE 16:0 and LPE 18:1) were purchased from Avanti Polar Lipids Inc., Alabaster, AL, USA.

### 2.2. Preparation of Rice Samples

All rice samples were ground to flour using a ball mill (Mixer Mill, MM301, Retsch GmbH & Co., Haan, Germany) by grinding six times at 30 rps for 30 s, as modified from Liu et al. [22]. Particle size of ground rice flour was analyzed using a Malvern Morphologi G3 (Morphologi G3, Malvern Panalytical Ltd., Malvern, UK) particle size analyzer.

### 2.3. Apparent Amylose Content of Fresh and Defatted Rice Flour and Lipid-Bound Amylose

Rice samples (20 mg) were defatted by heating with 5 mL of 85% ethanol at 60 °C for 30 min [23]. After cooling to room temperature, the sample was centrifuged at 1912× *g* (Sigma 4K-15, Sigma Laborzentrifugen, Osterode am Harz, Germany) for 10 min. The supernatant was discarded and the residual rice defatted by repeating the procedure once. Defatted rice flours were dried under low pressure.

Apparent amylose content of fresh (AAC) and defatted (AAC-L) rice flour was measured by iodine staining method modified from Blazek et al. [23]. A total of 4 mL of Milli-Q water and 2 mL of 1 M NaOH were added to 20 mg of rice flour in a 15 mL culture tube and heated for 30 min at 100 °C. Following this, 100 µL sample suspension was added to 5 mL of 0.5% trichloroacetic acid and mixed. Iodine solution (50 µL) (1.27 g of I_2_ and 3.0 g of KI in 1 L of water, 5 mM I_2_ solution) was added for staining and then mixed [24]. The stained solution (150 µL) was transferred to a 96-well flat-bottom plate (Greiner Bio-One GmbH, Frickenhausen, Germany), and the absorbance was measured at 620 nm using a colorimeter (KC4 multi-detection microplate reader, Bio Tek Instruments, Inc., Winooski, VT, USA). Rice flours from a previous study with known AAC were used to construct the standard curve and calculate AAC [25]. Lipid-bound amylose content was calculated by subtracting AAC from AAC-L.

### 2.4. Extraction and Analysis of Non-Starch and Starch Surface Rice Lipids

Rice lipids were extracted with water-saturated butanol (WSB) using a modified method of Geng et al. [26]. Rice flour (40 mg) was weighed in triplicate and added to 1.6 mL of WSB [26], sonicated for 40 min (Soniclean, Adelaide, SA, Australia) and centrifuged at 1311× *g* for 5 min (Sigma Laborzentrifugen, Osterode am Harz, Germany). Around 1 mL of the supernatant was collected in a 2 mL screw-cap HPLC vial (Agilent, Santa Clara, CA, USA) for lipid analysis and stored at −20 °C until analysis.

For analysis of rice lipids by LC-MS, an Agilent HPLC (series 1290) equipped with binary pump, auto-injector, vacuum degasser and diode array detector (DAD) coupled with Agilent quadrupole mass detector (MSD, 6120) was used. An Ascentis^®^ Express RP amide column (2.7 µm; 50 × 2.1 mm internal diameter, Supelco, Bellefonte, PA, USA) with 30% isopropanol +20% methanol +50% Milli-Q water with 10 mM ammonium formate (solvent A) and 75% isopropanol +20% methanol +5% Milli-Q water with 10 mM ammonium formate (solvent B) were used, and the solvent gradient applied is listed in Table 3. An electrospray ionization (ESI) ion source was used in mass detection ranging from 100 to 1200. Twenty-five orthogonally selected MS conditions (Table 4) were trialled considering drying gas flow, nebulizer pressure, drying gas temperature, capillary voltage and fragmentor. Two reproducible methods (Table 5) were developed using these selected MS conditions and using single-ion monitor (SIM) mode for four available mass selective detector (MSD) signal channels. Eight TAGs and four DAGs and four phosphatidylcholines (PCs) were analyzed using these two methods (Table 5). The column temperature was kept constant at 40 °C, and flow rate was 0.2 mL/min.

Commercial lipid standards were used for quantification of rice lipids (Table 2). Standard TAG 54:3 and DAG 36:4 were used for quantification of TAGs and DAGs, respectively, in Method 1. Standard TAG 54:6 was used for quantification of TAGs, and PC 36:2 and PC 34:1 were used for the quantification of PCs in Method 2. Standard lipids were prepared at a concentration of 160, 800, 4,000, 20,000, 100,000 and 200,000 nM by dissolving in WSB. Standard curves were constructed by plotting area under peak from chromatogram against concentration. The amount of lipids was calculated based on the standard curves.

### 2.5. Extraction and Analysis of Lysophospholipids (Starch Lipids)

Lysophospholipids (LPLs) were extracted from 16 mg of rice flour. The flour was first dried in a 2 mL screw-cap glass vial under vacuum and then extracted with 0.8 mL of 75% *n*-propanol for 2 h with heating at 100 °C using a dry block heater (Ratek Instruments Pty Ltd., Boronia, Victoria, Australia) [22], cooled to room temperature and centrifuged for 5 min at 1311× *g* (Sigma 2-5, Sigma Laborzentrifugen, Osterode am Harz, Germany). The supernatant (~0.4 mL) was collected in a 2 mL screw-cap glass vial for LC-MS analysis.

Rice flour LPLs were analyzed following the LC-MS protocol described by Liu et al. using LC-MS instrument as specified in Section 2.4 [22]. An Eclipse Plus C18 RRHD column (1.8 μm; 50 × 2.1 mm internal diameter, Agilent Technologies, Santa Clara, CA, USA) with flow rate 0.3 mL/min, column temperature 40 °C, was used with ESI ion source at scan mass range: 100–1200, capillary voltage: 3000 V (positive), drying gas flow: 12 L/min, drying gas temperature: 350 °C and nebulizer pressure: 35 psig (Table 6). The mobile phases were Milli-Q water with 0.005% trifluoroacetic acid (TFA) and acetonitrile with 0.005% TFA with a solvent gradient from 10 to 99% acetonitrile for 0–10 min, holding 99% acetonitrile for 1.5 min and returning to 10% acetonitrile in 1.5 min, which was maintained at 10% until 15 min. The injection volume was 3 µL, LPLs were detected in SIM mode (Table 6), and data were analyzed using ChemStation software [27].

### 2.6. Rice Lipid Removal and Addition

Rice lipids were removed or added to Doongara rice flour to confirm the effects of individual lipid classes on in vitro rice starch digestibility. Water-saturated butanol (WSB) extracts most types of the rice lipids, although not exhaustively [17]. For this reason, lipids from 200 mg of Doongara rice flour were extracted three times using 8 mL of WSB as described in Section 2.4, dried under low pressure and labelled as R − L. Four mL of WSB-extracted lipids from 200 mg of fresh rice flour was added to 100 mg of rice flour and labelled as R + L. A total of 1 mg each of TAG 54:6, DAG 36:4 and PC 36:2 was dissolved in WSB and LPC 16:0 in 75% *n*-propanol. Lipids were added to 100 mg of rice flour separately [28], dried under vacuum to constant weight and labelled as R + TAG, R + DAG, R + PC and R + LPC, respectively. These lipids were selected as a representative of each group of tested lipid class considering their abundance in rice flour and commercial availability. Doongara rice flour (R) and Doongara rice flours added to WSB (R + WSB) or 75% *n*-propanol (R + PPL) were dried under vacuum and used as controls.

### 2.7. In Vitro Starch Digestion of Rice Samples

The in vitro starch digestion method of rice samples was developed and optimized in our previous report [29], which contains detailed information about selection of digestive enzymes and glucometers. In brief, 15 mg of rice flours in triplicate were cooked with 60 µL of Milli-Q water for 20 min at 100 °C and cooled to room temperature [29]. Phosphate buffer (500 µL, pH 6.9) was added to the cooked rice and homogenized, from which 500 µL was further diluted with 1080 µL of phosphate buffer (pH 6.9) and digested with rat intestinal acetone powder for three hours. The released glucose was estimated by FreeStyle Optium Neo glucometer (Abbott Diabetes Care Ltd., Witney, UK), and rice starch digestibility is represented as mg glucose released per 100 mg dry rice flour (glucose concentration) or area under curve (AUC).

### 2.8. Data Analysis

Data are represented as mean ± standard deviation on dry weight basis. Area under curves from digestion time (minute) vs. mg glucose released/100 mg dry rice flour (AUC) were calculated using the trapezoid rule. General analysis of variance (ANOVA) and Duncan’s multiple range test at the *p* < 0.05 confidence level were carried out using GenStat 64-bit Release 18.1. Pearson’s correlation coefficient was calculated by Microsoft Excel 2016.

## 3. Results and Discussion

### 3.1. In Vitro Rice Starch Digestibility

**Figure 1:**
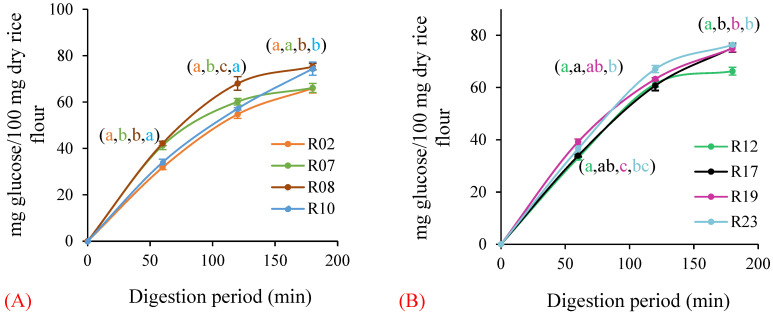
The highest and lowest in vitro starch digestibility among rice samples. (**A**) For the purchased commercial rice samples, R02, R07, R08 and R10; (**B**) for rice samples, R12, R17, R19 and R23 from Bangladesh. Error bars represent standard deviation of three replicates. min: minute. The different letters (a, b, c) denote a significant difference (*p* < 0.05) by Duncan’s multiple range test. The letter ab denotes no significant difference from a or b. The letter bc denotes no significant difference from b or c.

Since the variation in digestibility, amylose content and lipids was obviously different (see below) between the purchased commercial rice samples (R01–R10) and the rice samples acquired from Bangladesh (R11–R25), the results for these rice samples are presented separately. The rice samples exhibited a wider range of in vitro starch digestibility, with larger variations between the purchased commercial rice samples (R01–R10, Table 7 and Figure 1A) than those of rice samples acquired from Bangladesh (R11–R25, Table 7 and Figure 1B). Among the commercial rice samples, R02 (Doongara) had the lowest AUC and R08 (medium grain) had the highest AUC after 180 min of starch digestion (Figure 1A). Among rice samples from Bangladesh, R12 had the lowest AUC and R23 the highest AUC after 180 min of in vitro starch digestion (Figure 1B). The circle equivalent (CE) diameter of rice flours was around 11.86–17.46 μm, and the moisture content of rice flours was around 10.5–14.7%. In vitro starch digestibility had no correlation (R^2^ ≤ 0.02) with factors such as the particle size and moisture content of rice flour [30].

### 3.2. Association of Apparent Amylose Content of Rice Flour with In Vitro Starch Digestibility

**Figure 2:**
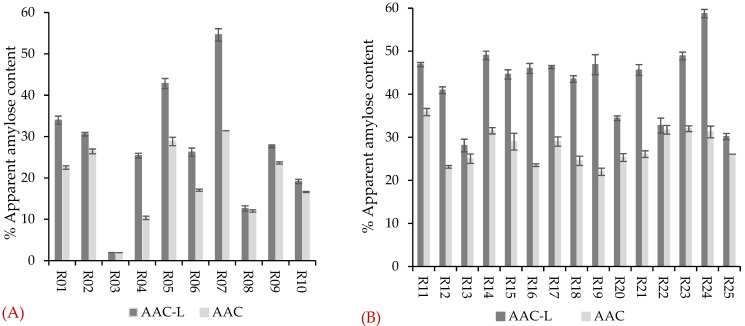
Apparent amylose content of rice samples (AAC) and defatted rice samples (AAC-L). (**A**) For the purchased commercial rice samples (R01–R10); (**B**) for the rice samples acquired from Bangladesh (R11–R25); error bars represent the standard deviation of triplicates. AAC-L: apparent amylose content in defatted rice; AAC: apparent amylose content.

**Figure 3:**
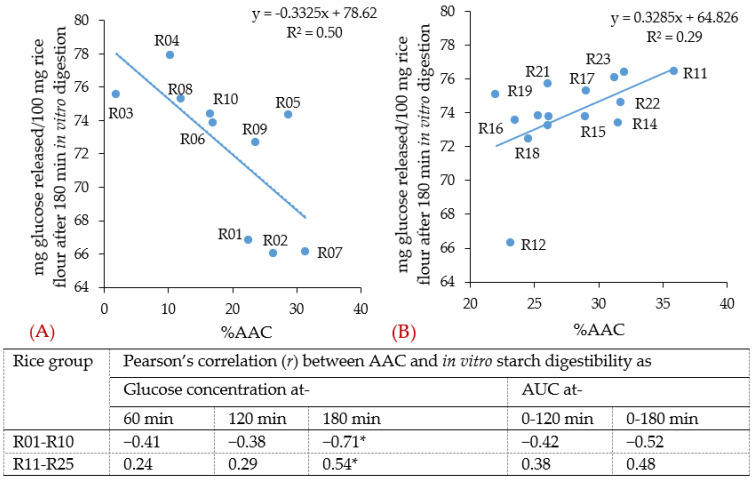
Correlation between apparent amylose content (AAC) and in vitro starch digestibility in rice. (**A**) For the purchased commercial rice samples (R01−R10); (**B**) for the rice samples acquired from Bangladesh (R11−R25); *: significant at *p* < 0.05; min: minute.

In this study, the apparent amylose content (AAC) in all rice samples ranged from 1.9% to 35.9% (Figure 2), with a larger difference (from 1.9% to 31.4%, Figure 2A) between the purchased commercial samples than (22.0% to 35.9%, Figure 2B) between the samples acquired from Bangladesh [30]. When considering all the rice samples together, no correlations could be identified between the rice flour digestion and AAC. However, when considering the purchased commercial rice samples alone, the AAC had a weak negative correlation (*R*^2^ = 0.50 and *p* < 0.05) with the glucose concentration after 180 min of digestion (Figure 3A). On the other hand, when considering the rice samples acquired from Bangladesh alone, the AAC had a weaker positive correlation (*R*^2^ = 0.29 and *p* < 0.05) with the glucose concentration after 180 min of digestion (Figure 3B). In previous studies, a negative correlation of AAC with starch digestibility was detected among rice samples containing a wide range of AAC [31,32,33,34], but not in intermediate-to-high apparent amylose-containing rice samples [35,36]. The correlation between the AAC and starch digestibility may depend on the rice gelatinisation properties during cooking and binding of amylose with other rice components [30,37]. Around 10 g of lipids is needed to form a complex with 100 g of amylose, and excess lipids or amylose may remain unbound [38]. It is possible in high-AAC rice that the amylose/lipid ratio is larger than 10 and more amylose may remain unbound. The presence of unbound amylose may be the cause of the positive correlation between AAC and in vitro starch digestion (Figure 3B) [30].

The negative correlation between AAC and starch digestibility was only significant at 180 min for the purchased commercial rice samples (Figure 3A, R01–R10). The difference among the glucose concentration at 180 min might be due to the carbohydrate content of the rice samples as the glucose released was calculated based on rice flour in this study. The “mg carbohydrate/100 mg rice” of the purchased commercial rice samples was slightly different, between 76.4 and 79.6 (information on the packages). However, there were no correlations between the glucose concentration at 180 min and the carbohydrate content of these rice samples.

### 3.3. LC-MS Analysis of Rice Lipids

Gas chromatography (GC) and thin-layer chromatography (TLC) have been widely used for the analysis of fatty acid composition and characterization of lipids in rice grain [39,40]. Lipid analysis by GC requires breaking down the original lipid molecule and methylating the released fatty acids to methyl ester form (FAMEs) [41]. For this reason, GC can only reveal the fatty acid composition but not the original lipid species such as phospholipids and triacylglycerols (TAGs). TLC can visualize the lipid species, but the amount of the lipids cannot be accurately quantified. A combination of TLC and GC can quantify the individual lipid species; however, it would be extremely time-consuming [39,40].

Liquid chromatography–mass spectrometry (LC-MS) and liquid chromatography– tandem mass spectrometry (LC-MS/MS) can identify and quantify individual plant lipids directly after solvent extraction [22,42]. LC-MS has been used for rice and wheat grain lysophospholipid (LPL) analysis [22,27] and TAG analysis in maize, rapeseed and sunflower oil [43]. Sphingolipids have been characterized from rice leaves and roots using LC-MS/MS [42], and untargeted LC-MS/MS has analyzed lipids in wheat grain [26]. The current study attempted to quantify all the major original acylglycerols, such as TAGs, diacylglycerols (DAGs) and phosphatidylcholine (PCs), in rice grain by LC-MS.

**Figure 4:**
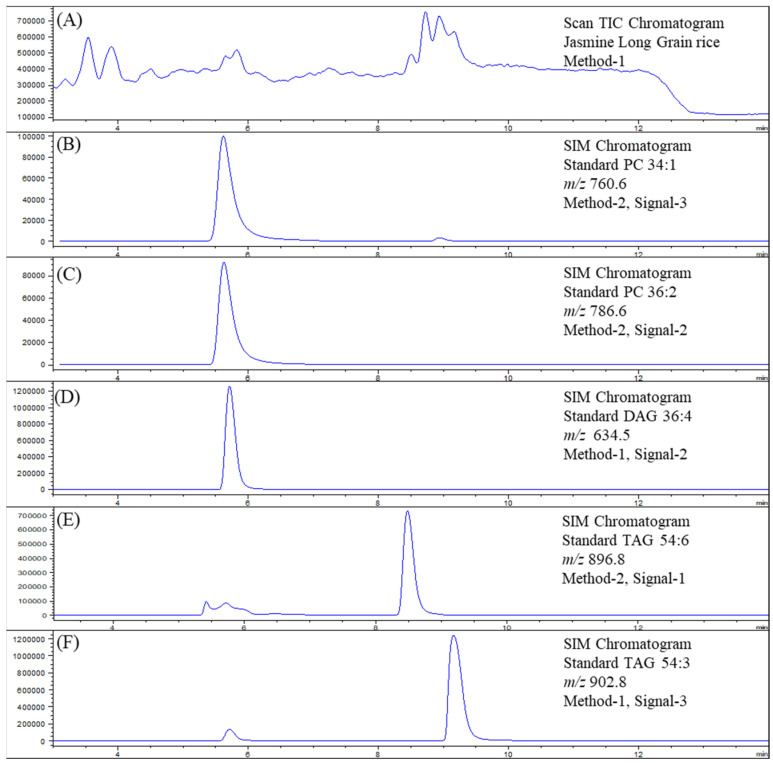
LC-MS chromatogram for rice lipid analysis (R04, Jasmine long-grain rice). Total ion chromatogram (TIC) of the rice lipid in Method 1 scan (**A**). Single-ion monitoring (SIM) for individual standards, PC 34:1 (**B**); PC 36:2 (**C**); DAG 36:4 (**D**); TAG 54:6 (**E**); TAG 54:3 (**F**). The information about method and signal is given in Table 5.

**Figure 5:**
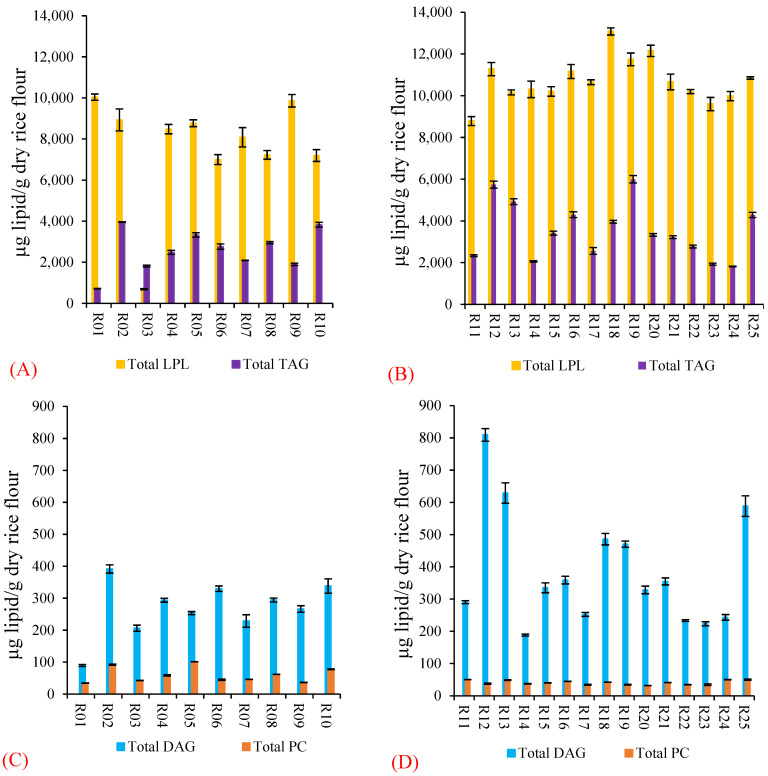
Differences in rice lipids among the rice samples. LPL: lysophospholipid; TAG: triacylglycerol; DAG: diacylglycerol; PC: phosphatidylcholine. (**A**) Total LPLs and total TAGs for purchased commercial samples R01–R10; (**B**) total LPLs and total TAGs for the samples acquired from Bangladesh, R11–R25; (**C**) total DAGs and total PCs for purchased commercial samples R01–R10; (**D**) total DAGs and total PCs for the samples acquired from Bangladesh, R11–R25.

Eight TAGs, four DAGs and four PCs from WSB extracts of rice flours were analyzed by the two optimized LC-MS methods. However, as the elution times of lipids overlapped, the lipids were separated into two groups and quantified (Figure 4). It should be noted that Method 2 could not detect DAGs but was more sensitive for PCs. The LC-MS methods were reproducible (Figure 5) and efficient, and the trace amount of lipids such as PCs could be quantified directly from the solvent extracts of small amounts of rice flour (less than 50 mg).

TAGs were the major non-starch and starch surface lipid (713–5998 µg/g dry rice flour, Figure 5A,B) along with DAGs (90–809 µg/g dry rice flour, Figure 5C,D) and PCs (32–101 µg/g dry rice flour, Figure 5C,D). TAG 52:3 was the most abundant of eight TAGs except in three rice samples (R03, R14 and R24). TAG content was the highest in R19 and the lowest in R01 (Basmati) (Figure 5A,B). Previously, TAGs were fractionated by TLC then analyzed by GC, and all TAGs reported in this study were found in rice flour except TAG 54:3 [39].

DAG content was highest in R12 and lowest in R01 (Basmati) (Figure 5C,D). Total PC content was significantly different among rice samples, and values were higher in purchased commercial rice samples (R01–R10) than the rice samples acquired from Bangladesh (R11–R25). PC was highest in R05 (Kalijeera) and lowest in R20 (Figure 5C,D). The individual PCs (PC 34:1, PC 34:2, PC 36:2 and PC 36:4) characterized in this study have been reported in brown rice [40].

Although WSB can extract lysophospholipid (LPLs), heating the rice flour to 100 °C in 25% water and 75% *n*-propanol solution allows more LPLs to be extracted [17,44]. Five lysophosphatidylcholines (LPCs), namely LPC 14:0, LPC 16:0, LPC 18:1, LPC 18:2 and LPC 18:3, and five lysophosphatidylethanolamines (LPEs), namely LPE 14:0, LPE 16:0, LPE 18:1, LPE 18:2 and LPE 18:3, were estimated (Table 8). Total LPL content was 687–13,081 µg LPLs/g dry rice flour in rice samples (Figure 5A,B). R18 had the highest LPL content and R03 (glutinous rice) had the lowest amount of LPLs (Figure 5A,B). LPC 16:0 was highest, and LPE 18:3 was lowest among the rice LPLs measured, which agreed with previous studies [45]. LPL was the major class of lipids in milled rice (Figure 5A,B), which was also evident in a previous study [46].

### 3.4. Association between Native Lipid Content and In Vitro Rice Starch Digestibility

The association between individual native lipid content and in vitro rice starch digestibility could not been seen when all the rice samples used in this study were considered together. This could be due to the similar digestibility among all rice samples and low contents of lipids in milled rice. The fact is that there were so many differences in the major components, starch and protein, between the rice samples, which could also affect rice flour digestibility [2,30]. The effects from other major components could make it difficult to find the association between rice lipids, a minor component, and rice flour digestibility. For example, the large variation in the amylose content of some rice samples (Figure 2) alone could have caused significant differences in the rice flour digestion. Therefore, rice samples were separated into two groups, and the association study was attempted. The first group was purchased commercial rice samples (R01–R10) with large variation in the AAC (Figure 2A). The second group (R11–R25) was the rice samples acquired from Bangladesh with a relatively similar AAC (Figure 2B).

The addition of TAGs containing vegetable oil and animal fat to rice decreases in vitro rice starch digestibility [10]. However, there was no correlation between individual TAGs and DAGs and in vitro starch digestibility in the purchased commercial rice samples (R01–R10). Because a low AAC may play a significant role in rice in vitro digestibility, R03 (a glutinous rice) was excluded from association analysis of all types of lipids with in vitro starch digestibility. Following exclusion of R03 from the analysis, some negative correlations (−0.54 ≤ *r* ≤ −0.42; *p* > 0.05) were observed between TAGs and glucose concentration at 60 min (R01, R02, R04–R10) (Table 9). There were negative correlations between individual TAGs and DAGs and in vitro starch digestion in the rice samples acquired from Bangladesh (R11–R25) (Table 9). The negative correlation between individual TAGs and the glucose concentration at 180 min of in vitro digestion was significant (−0.69 ≤ *r* ≤ −0.52; *p* < 0.05) in the rice samples acquired from Bangladesh (R11–R25) except for TAG 50:2 and TAG 52:2 (*r* = −0.51 and −0.50) (Table 9).

The negative correlations between individual DAGs and glucose concentration at 180 min of in vitro starch digestion were significant (*p* < 0.01) in the rice samples acquired from Bangladesh (R11–R25), but there were no correlations in the purchased commercial rice samples (R01, R02, R04–R10). Individual PC content had weak negative correlations with glucose concentration at 60 and 120 min of in vitro starch digestion in the purchased commercial rice samples (R01, R02, R04–R10) but no correlation with glucose concentration at 180 min digestion (Table 9). However, there was no correlation between individuals or total PCs with in vitro starch digestibility among the rice samples acquired from Bangladesh (R11–R25) (Table 9). Most of the individual LPCs, LPEs and the total LPL content were negatively correlated with in vitro starch digestibility of rice samples (Table 9).

The correlation of individual rice lipids with in vitro rice starch digestibility has not been previously analyzed. TAGs (TAG 54:6, TAG 54:4, TAG 54:3 and TAG 54:1) and DAGs (DAG 36:4, DAG 36:3 and DAG 36:2) with longer fatty acid chains had negative correlations with glucose concentration after 180 min of digestion to a larger extent than TAG 50:2, TAG 52:2, TAG 52:3 and DAG 34:2 (−0.50 ≤ *r* ≤−0.69) with shorter fatty acid chains in rice samples from Bangladesh (Table 9, R11–R25). In a previous study, addition of long-chain monoacylglycerols reduced in vitro starch digestibility more than short-chain monoacylglycerols [47]. However, such correlations between the chain length of acyl fatty acids and the AUC at 180 min of digestion were not obvious for LPLs (Table 9).

Negative correlations of individual lipids with in vitro starch digestibility suggest rice lipids decrease starch digestibility (Table 9). Starch–lipid interactions have been assumed to be a cause of delayed in vitro starch digestion in rice samples when non-rice lipids were added during rice heat-moisture treatment [10,11]. Addition of polar lipids such as monoacylglycerol and free fatty acids formed crystalline starch (V-type) in maize [48], which might be more resistant to digestion [49]. Since LPLs are a major polar lipid in polished rice (Figure 5A,B), they may play an important role in rice starch digestion by a different mechanism than that of the major non-polar rice lipids TAGs.

It can be hard to find the relationship between native rice lipids and rice flour digestibility by association study alone as the rice samples used for most of the studies would have a large variation in other components, also affecting rice digestibility. We recently researched genetically modified rice with only the rice lipid composition changed by the FAD2-1 gene [50]. This genetically modified rice sample only had a difference in lipids from the non-modified rice sample, and the change in the lipid composition alerted the starch swelling power [50], which would affect the rice digestibility. Future research on the genetically modified rice sample for lipid composition could provide a better understanding on the effects of rice lipids on rice digestion.

### 3.5. Impact of Lipid Removal and Addition to Rice Flour on In Vitro Starch Digestibility

**Figure 6:**
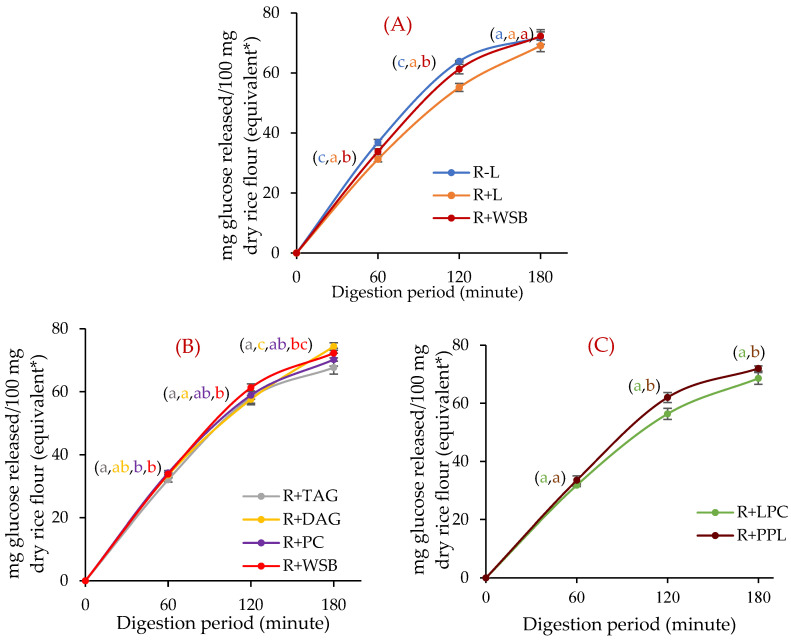
In vitro rice starch digestibility affected by lipid removal and addition to rice flour (Doongara, R02). (**A**) Lipid addition (R + L) to rice flour and removal (R − L) from rice flour; (**B**) addition of TAG 54:6 (R + TAG); DAG 36:4 (R + DAG); and PC 36:2 (R + PC); and (**C**) LPC 16:0 addition (R + LPC). R + WSB: water-saturated-butanol-treated control rice flour; R + PPL: 75% *n*-propanol-treated control rice flour. Error bars represent standard deviation of three replicates. * Equivalent to the weight of untreated rice flour. The different letters (a, b, c) denote a significant difference (*p* < 0.05) by Duncan’s multiple range test. The letter ab denotes no significant difference from a or b. The letter bc denotes no significant difference from b or c.

**Figure 7:**
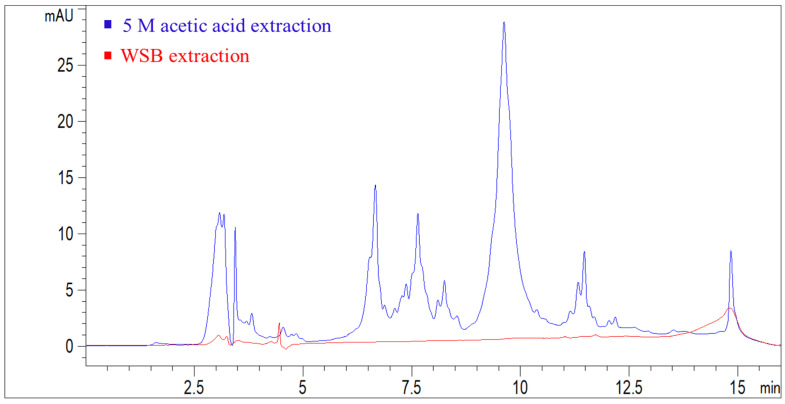
Comparative HPLC chromatogram from UV absorbance at 280 nm of rice protein extract by 5 M acetic acid and rice lipid extract by water-saturated butanol (WSB). Rice sample: Doongara (R02); the detailed method for rice protein extraction and analysis can be found in our previous publication [30].

The commercial Doongara rice (Table 1, R02), an Australian-bred low-GI rice, was used in the study to evaluate the effect of adding or removing lipids on in vitro rice starch digestibility. Removal of native lipids from rice flour by water-saturated butanol (WSB) (R − L) increased in vitro starch digestibility (Figure 6A) while addition of WSB-extractable native lipids to rice flour (R + L) decreased in vitro starch digestibility (Figure 6A). These two experiments confirmed the role of rice lipids in decreasing in vitro starch digestibility. Some protein may be removed from rice flour during defatting of rice flour [51], which might affect starch digestibility. The WSB supernatant from defatted rice flour, analyzed by the HPLC method, showed no rice protein was extracted during defatting (Figure 7) [30].

Addition of 1% of individual lipids TAG 54:6 (R + TAG), DAG 36:4 (R + DAG), PC 36:2 (R + PC) and LPC 16:0 (R + LPC) to rice flour decreased in vitro rice starch digestibility (Figure 6B,C). The degree of in vitro starch digestibility decreased upon addition of individual lipids was in the order of: LPC 16:0 > TAG 54:6 > PC 36:2 > DAG 36:4. To offset the effects of adding solvents to rice flour, the in vitro starch digestibility of R + TAG, R + DAG and R + PC were compared against control rice samples treated with WSB (R + WSB), and R + LPC was compared against the control rice sample treated with 75% *n*-propanol (R + PPL). There was no significant (*p* > 0.01) difference in AUCs at 120 and 180 min of digestion among R + WSB, R + PPL and fresh control rice flour (R) (Table 10).

In this study, the addition of TAG 54:6 and DAG 36:4 decreased starch digestibility; the AUC of 1%-TAG 54:6-treated rice flour decreased by 5.9% after three hours of digestion in comparison to WSB-treated control rice flour (Figure 6B). TAGs were the major rice non-starch lipids relative to DAGs (Figure 5) [17], and addition of DAG 36:4 to rice flour did not significantly reduce in vitro starch digestibility (*p* > 0.05). Addition of 1% LPC 16:0 to rice flour reduced glucose release following three hours of in vitro starch digestion by 7.4% (Figure 6C). Since LPE 16:0 has a similar structure to LPC 16:0, adding LPE 16:0 could also significantly affect the in vitro starch digestion, which should be addressed in future studies.

### 3.6. Possible Mechanism of the Impact of Rice Lipids on In Vitro Rice Starch Digestibility

**Figure 8:**
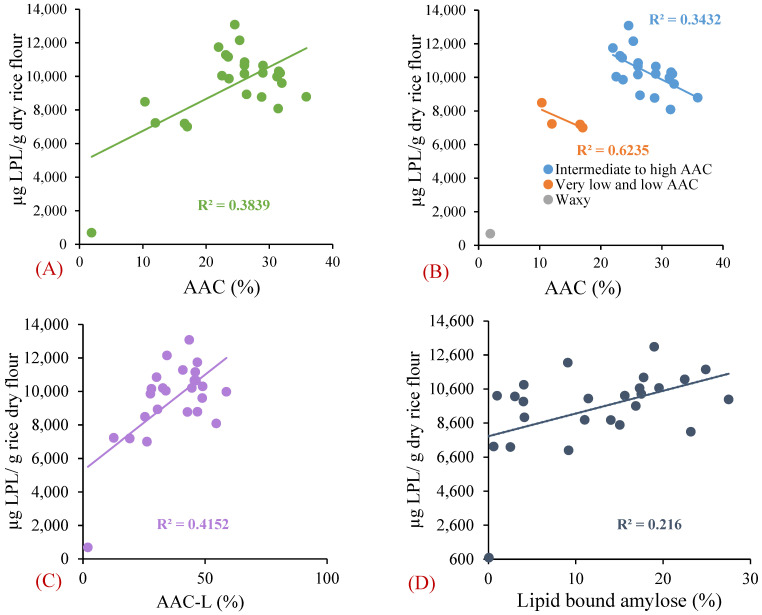
Correlation of lysophospholipid (LPL) content with (**A**) apparent amylose content (AAC); (**B**) low AAC, and intermediate and high AAC; (**C**) AAC in defatted rice flour (AAC-L); and (**D**) lipid-bound amylose.

Rice lipids may bind with amylose and affect starch digestion. The difference in amylose content between defatted and un-defatted rice flour is indicative of the lipid-bound amylose content [23]. The AAC of defatted rice (AAC-L) was higher than the AAC of non-defatted samples (Figure 2), suggesting there was lipid–amylose complex formation in the rice [23]. There was a weak positive correlation (*R*^2^ = 0.38) between AAC and total LPL content (Figure 8A), which is in line with previous reports [52]. When the low-apparent-amylose-containing rice samples were grouped separately from intermediate- and high-apparent-amylose-containing rice samples, negative correlations (*R*^2^ = 0.62 in low-AAC and *R*^2^ = 0.34 in intermediate- and high-AAC rice) were observed between the AAC and LPL content (Figure 8B). On the other hand, AAC-L (*R*^2^ = 0.42) and lipid-bound amylose (*R*^2^ = 0.22) had weak positive correlations with LPL content in rice samples (Figure 8C,D).

**Figure 9:**
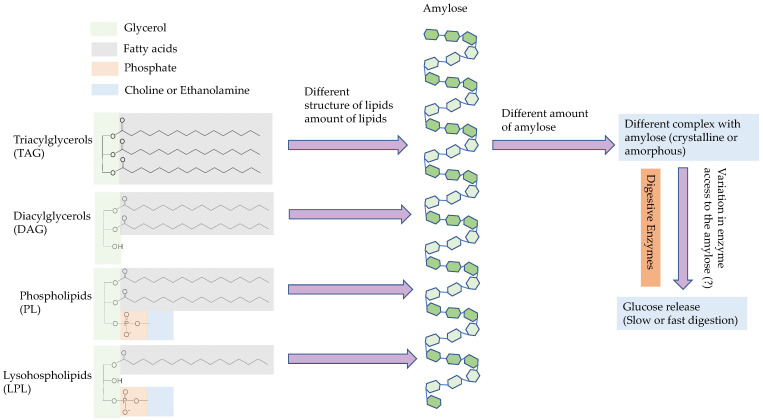
Illustrated speculation for the possible mechanism of the rice lipids’ impact on rice starch digestibility.

There was no correlation between lipid-bound amylose (or the amylose–lipid complex) and glucose concentration during in vitro rice starch digestion (−0.02 ≤ *r* ≤ 0.24) (Table 11). Starch–lipid complexes may be amorphous (Type-I) or crystalline (Type-II) [53,54], and the crystalline starch–lipid complex can inhibit starch digestibility to a greater extent than the amorphous starch–lipid complex [13]. The degree of V crystallinity is negatively correlated with in vitro starch digestibility in brown rice [49], and so it may be important to understand the amount of lipids participating in the formation of crystalline or amorphous complexes, which could have different effects on starch digestibility(Figure 9).

## 4. Conclusions

The current study demonstrated for the first time the negative impact of individual native rice lipids on in vitro rice starch digestibility. Most of the tested TAGs, DAGs, PCs and LPLs were negatively associated with in vitro rice starch digestion either in the early or late stage of digestion. However, the association study was not conclusive, possibly due to the large effects of other rice grain components (e.g., amylose content). Genetically modified rice with differences only in rice lipids should be used in the future to understand the effects of rice lipids on rice starch digestion.

Removal of lipids from rice flour increased in vitro starch digestibility while addition of extracted lipids to rice flour reduced starch digestion. Unlike in previous studies, the added and removed lipids were the same lipids in milled rice. Addition of triacylglycerol (TAG 54:6), diacylglycerol (DAG 36:4), phosphatidylcholine (PC 36:2) and lysophosphatidylcholine (LPC 16:0) reduced starch digestibility, and the effects were more pronounced with addition of LPC 16:0 and TAG 54:6. This study suggests lipids of rice grains can affect the rice starch digestibility, and modifying the lipid content and composition in rice by breeding may assist in achieving desirable rice starch digestibility.

## Figures and Tables

**Table 1:** Rice samples used in this study.

Rice Sample	Growing Location	Grain Type	Rice Name
R01	Pakistan	Long, thin	Basmati
R02	Australia	Long	Doongara
R03	Australia	Short	Glutinous
R04	Thailand	Long	Jasmine
R05	Bangladesh	Short	Kalijeera
R06	Australia	Short	Koshihikari
R07	Australia	Long	Long grain
R08	Australia	Medium	Medium grain
R09	Australia	Long	Long grain
R10	Australia	Short	Sushi
R11	Bangladesh	Long, thin	ND
R12	Bangladesh	Medium, thin	ND
R13	Bangladesh	Medium	ND
R14	Bangladesh	Medium, bold	ND
R15	Bangladesh	Medium	ND
R16	Bangladesh	Long, non-sticky	ND
R17	Bangladesh	Long, thin	ND
R18	Bangladesh	Short	ND
R19	Bangladesh	Medium, thin, non-sticky	ND
R20	Bangladesh	Medium, thin	ND
R21	Bangladesh	Medium, thin	ND
R22	Bangladesh	Short, medium bold	ND
R23	Bangladesh	Medium, bold	ND
R24	Bangladesh	Medium, bold	ND
R25	Bangladesh	Short	ND

ND: The identity of the rice collected from Bangladesh has not been disclosed to avoid potential conflict of interest.

**Table 2:** List of lipid standards used in experiments.

Lipid Type	Lipid	Product Code
Triacylglycerols	Glyceryl trioleate (TAG 54:3)	T7140 ^a^
	Glyceryl trilinoleate (TAG 54:6)	T9517 ^a^
Diacylglycerols	1,3-dilinoleoyl-*rac*-glycerol (DAG 36:4)	D9508 ^a^
Phospholipids	1,2-dioleoyl-*sn*-glycero-3-phosphocholine (PC 36:2)	P6354 ^a^
	2-oleoyl-1-palmitoyl-*sn*-glycero-3-phosphocholine (PC 34:1)	42773 ^a^
Lysophospholipids	1-palmitoyl-2-hydroxy-*sn*-glycero-3-phosphocholine (LPC 16:0)	855675 ^b^
	1-oleoyl-2-hydroxy-*sn*-glycero-3-phosphocholine (LPC 18:1)	845875 ^b^
	1-palmitoyl-2-hydroxy-*sn*-glycero-3-phosphoethanolamine (LPE 16:0)	856705 ^b^
	1-oleoyl-2-hydroxy-*sn*-glycero-3-phosphoethanolamine, (LPE 18:1)	846725 ^b^

^a^ purchased from Sigma-Aldrich Co., St. Louis, MO, USA. ^b^ purchased from Avanti Polar Lipids Inc., Alabaster, AL, USA.

**Table 3:** LC-MS solvent gradient for the analysis of rice non-starch and starch surface lipids.

Time (min)	% Solvent A	% Solvent B
0	95	5
2	60	40
3	40	60
4	20	80
5	20	80
6	10	90
7	10	90
8	0	100
11	0	100
11.5	95	5
14	95	5

Solvent A: 30% isopropanol +20% methanol +50% Milli-Q water with 10 mM ammonium formate. Solvent B: 75% isopropanol +20% methanol +5% Milli-Q water with 10 mM ammonium formate.

**Table 4:** Orthogonally selected mass spectrometry (MS) conditions used in the method development for rice lipid analysis.

Orthogonal Trial No.	Drying Gas Flow (L/min)	Nebulizer Pressure (psig)	Drying Gas Temperature (°C)	Capillary Voltage (V)	Fragmentor
01	5	35	350	1000	250
02	5	25	200	3000	100
03	9	45	300	2000	250
04	7	35	200	2000	100
05	9	55	200	1000	150
06 (Method 2)	5	25	250	2000	350
07	9	25	350	4000	100
08	3	55	250	3000	250
09	11	45	350	3000	100
10	7	55	350	1000	350
11	3	45	200	4000	350
12	7	45	250	1000	100
13	9	25	250	1000	100
14	3	25	350	2000	150
15	11	25	200	1000	250
16	3	35	300	1000	100
17	9	35	200	3000	350
18	11	35	250	4000	150
19	5	45	200	1000	150
20	7	25	300	3000	150
21	5	55	300	4000	100
22	11	55	200	2000	100
23 (Method 1)	7	25	200	4000	150
24	11	25	300	1000	350
25	3	25	200	1000	100

**Table 5:** MS conditions for two LC-MS methods used in non-starch and starch surface rice lipids analysis.

Signal	General Condition: Gain 1, Dwell 140 ms, Nebulizer Pressure 25 psig									
	Method 1: Drying Gas Flow 7 L/min, Drying Gas Temperature 200 °C, Capillary Voltage 4000 V (Positive), Fragmentor 150					Method 2: Drying Gas Flow 5 L/min, Drying Gas Temperature 250 °C, Capillary Voltage 2000 V (Positive), Fragmentor 350				
	Lipid	MW ^a^	Ion ^b^	*m/z* ^c^	RT ^d^	Lipid	MW	Ion	*m/z*	RT
1	DAG 34:2	592.0	M + NH_4_^+^	610.5	5.9	PC 36:4	782.0	M + H^+^	782.6	5.3
	TAG 50:2	830.0	M + NH_4_^+^	848.8	8.9	TAG 54:6	879.4	M + NH_4_^+^	896.8	8.5
2	DAG 36:4	616.5	M + NH_4_^+^	634.5	6.0	PC 36:2	786.1	M + H^+^	786.6	5.4
	TAG 54:4	882.8	M + NH_4_^+^	900.8	9.1	TAG 52:4	854.7	M + NH_4_^+^	872.7	8.5
3	DAG 36:3	618.5	M + NH_4_^+^	636.6	6.2	PC 34:1	760.1	M + H^+^	760.6	5.5
	TAG 54:3	885.4	M + NH_4_^+^	902.8	9.2	TAG 52:3	856.8	M + NH_4_^+^	874.8	8.9
4	DAG 36:2	620.6	M + NH_4_^+^	638.6	6.2	PC 34:2	757.6	M + H^+^	758.6	5.3
	TAG 54:1	888.8	M + NH_4_^+^	906.9	9.2	TAG 52:2	859.4	M + NH_4_^+^	876.7	9.3

^a^ MW: molecular weight; ^b^ Ion: MW + adduct ion; ^c^ *m*/*z*: mass-to-charge ratio; ^d^ RT: retention time in minutes.

**Table 6:** MS conditions for LC-MS method used in rice lysophospholipid analysis.

Signal	MS Condition: Drying Gas Flow 12 L/min, Drying Gas Temperature 350 °C, Capillary Voltage 3000 V (Positive), Nebulizer Pressure 35 psig, Fragmentor 150, Gain 1				
	Lipid	MW *	Ion **	*m/z* ***	RT (min) ****
1	LPC 18:3	517.6	M + H^+^	518.0	6.8
	LPC 18:2	519.7	M + H^+^	520.0	7.5
	LPC 18:1	521.7	M + H^+^	522.0	8.6
2	LPC 14:0	467.6	M + H^+^	468.0	6.5
	LPC 16:0	495.6	M + H^+^	496.0	8.6
3	LPE 18:3	475.6	M + H^+^	476.0	5.8
	LPE 18:2	477.6	M + H^+^	478.0	6.4
	LPE 18:1	479.6	M + H^+^	480.0	7.1
4	LPE 14:0	425.5	M + H^+^	426.0	5.8
	LPE 16:0	453.6	M + H^+^	454.0	6.9

* MW: molecular weight; ** Ion: MW + adduct ion; *** *m/z*: mass-to-charge ratio; **** RT: retention time in minutes.

**Table 7:** In vitro rice starch digestibility.

Rice Sample	Glucose Concentration at:			AUC at:	
	60 min	120 min	180 min	0–120 min	0–180 min
R01	34.5	54.2	66.8	61.5	122.0
R02	31.8	54.8	66.0	59.2	119.5
R03	40.6	60.8	75.5	70.9	139.1
R04	39.4	59.4	77.9	69.0	137.7
R05	35.8	58.0	74.3	64.8	130.9
R06	42.0	68.7	73.8	76.4	147.6
R07	41.3	60.2	66.1	71.4	134.5
R08	42.2	68.0	75.3	76.2	147.8
R09	36.7	59.4	72.7	66.4	132.4
R10	34.0	57.2	74.4	62.6	128.4
R11	37.6	64.7	76.4	69.9	140.5
R12	33.1	61.4	66.3	63.9	127.7
R13	35.2	60.2	73.2	65.2	131.9
R14	35.2	64.2	73.4	67.3	136.1
R15	34.7	64.0	73.7	66.7	135.5
R16	36.8	63.5	73.5	68.6	137.1
R17	33.9	60.8	75.2	64.3	132.3
R18	34.9	63.9	72.4	66.8	135.0
R19	39.2	63.3	75.0	70.9	140.1
R20	35.7	61.5	73.8	66.5	134.1
R21	35.9	64.8	75.7	68.3	138.6
R22	38.0	61.3	74.6	68.7	136.6
R23	36.6	67.1	76.4	70.1	141.9
R24	38.5	62.6	76.0	69.8	139.1
R25	37.2	59.6	73.8	67.0	133.7

AUC: area under curve from digestion time (minute) vs. mg glucose released/100 mg dry rice flour; (AUCs) were calculated using the trapezoid rule.

**Table 8:** Lysophosphatidylcholine (LPC) content of polished rice samples.

Rice Samples	Individual LPC Content (µg/g Dry Rice Flour)					Individual LPE Content (µg/g Dried Rice Flour)				
	LPC 18:3	LPC 18:2	LPC 18:1	LPC 14:0	LPC 16:0	LPE 18:3	LPE 18:2	LPE 18:1	LPE 14:0	LPE 16:0
R01	108 ± 1.6	3143 ± 46.5	622 ± 34.1	770 ± 11.1	4367 ± 128.2	5 ± 0.5	440 ± 1.9	75 ± 1.1	34 ± 1.4	475 ± 3.4
R02	96 ± 3.8	2685 ± 163.7	632 ± 23.2	466 ± 35.3	3891 ± 265.1	6 ± 0.4	493 ± 20.2	85 ± 4.0	28 ± 2.2	546 ± 21.3
R03	34 ± 1.2	134 ± 6.7	69 ± 1.3	52 ± 3.2	378 ± 18.8	NF	NF	NF	NF	19 ± 1.0
R04	102 ± 3.3	2674 ± 77.2	599 ± 14.8	526 ± 20.7	3613 ± 97.3	4 ± 1.0	401 ± 13.1	67 ± 1.0	25 ± 1.6	472 ± 10.7
R05	103 ± 4.7	2855 ± 68.2	897 ± 36.1	365 ± 18.6	3497 ± 95.6	6 ± 0.4	463 ± 10.7	125 ± 1.1	15 ± 0.6	446 ± 8.2
R06	102 ± 2.3	2034 ± 74.6	592 ± 37.5	413 ± 13.6	3064 ± 109.3	4 ± 0.3	326 ± 8.4	73 ± 3.0	19 ± 0.5	373 ± 9.2
R07	98 ± 4.9	2473 ± 161.9	445 ± 4.9	622 ± 38.2	3514 ± 262.0	4 ± 0.4	391 ± 17.5	59 ± 3.9	29 ± 1.4	439 ± 4.9
R08	83 ± 1.5	2199 ± 34.0	661 ± 62.0	320 ± 2.8	3098 ± 117.1	2 ± 0.0	349 ± 7.4	82 ± 3.5	15 ± 0.3	420 ± 10.4
R09	119 ± 3.9	3297 ± 107.7	460 ± 25.0	470 ± 31.4	4370 ± 129.2	8 ± 0.4	528 ± 20.0	53 ± 1.5	22 ± 1.4	534 ± 15.4
R10	110 ± 3.5	2128 ± 70.3	615 ± 38.6	475 ± 17.6	3031 ± 137.8	6 ± 0.7	353 ± 10.0	78 ± 3.6	24 ± 0.7	376 ± 10.8
R11	94 ± 1.5	2854 ± 59.9	525 ± 37.9	774 ± 9.1	3442 ± 107.6	5 ± 0.1	463 ± 5.7	79 ± 1.3	46 ± 1.4	502 ± 6.1
R12	94 ± 1.3	3851 ±55.9	685 ± 69.0	475 ± 6.3	4896 ± 203.8	5 ± 0.1	557 ± 3.6	91 ± 3.5	24 ± 0.4	601 ± 9.1
R13	106 ± 5.1	3482 ± 81.5	563 ± 44.2	470 ± 9.2	4419 ± 45.0	7 ± 0.1	455 ± 12.6	78 ± 1.3	23 ± 1.5	557 ± 12.9
R14	86 ± 3.4	3161 ± 118.7	388 ± 26.1	843 ± 66.8	4866 ± 190.3	4 ± 0.4	421 ± 15.1	53 ± 3.2	35 ± 1.4	448 ± 14.6
R15	98 ± 3.7	3258 ± 58.6	493 ± 35.8	722 ± 12.0	4586 ± 138.1	7 ± 0.1	489 ± 6.2	62 ± 2.2	34 ± 1.2	455 ± 4.7
R16	112 ± 3.8	3617 ± 112.6	567 ± 11.2	716 ± 30.4	4885 ± 161.8	8 ± 0.1	575 ± 17.1	80 ± 2.2	37 ± 1.6	566 ± 22.4
R17	91 ± 1.2	3431 ± 30.2	602 ± 22.7	952 ± 29.9	4343 ± 36.0	6 ± 0.1	492 ± 9.9	81 ± 1.6	56 ± 2.1	593 ± 13.9
R18	142 ± 5.5	4183 ± 24.6	788 ± 40.4	622 ± 7.3	5950 ± 87.5	10 ± 0.5	567 ± 7.6	96 ± 2.0	28 ± 0.6	696 ± 4.9
R19	145 ± 7.6	3926 ± 73.6	446 ± 15.3	1066 ± 45.1	5008 ± 157.1	11 ± 0.5	511 ± 16.3	55 ± 1.8	52 ± 2.0	526 ± 12.2
R20	99 ± 2.1	3654 ± 83.8	823 ± 46.6	1169 ± 25.4	5019 ± 90.7	7 ± 0.4	575 ± 15.9	114 ± 2.5	66 ± 4.3	623 ± 12.6
R21	128 ± 7.4	3347 ± 138.0	640 ± 25.8	681 ± 52.8	4809 ± 102.1	7 ± 0.4	411 ± 23.0	72 ± 3.8	34 ± 3.1	532 ± 27.2
R22	104 ± 1.0	3736 ± 29.5	313 ± 11.4	931 ± 8.8	4198 ± 41.6	6 ± 0.4	445 ± 3.7	34 ± 1.2	39 ± 1.3	394 ± 9.6
R23	92 ± 2.7	2957 ± 100.6	353 ± 1.8	777 ± 35.3	4468 ± 159.5	4 ± 0.2	452 ± 13.9	44 ± 1.7	33 ± 0.9	420 ± 16.1
R24	85 ± 1.1	3295 ± 95.6	300 ± 7.8	911 ± 24.1	4464 ± 86.3	3 ± 0.0	443 ± 6.5	37 ± 1.6	38 ± 0.9	405 ± 4.7
R25	131 ± 1.4	3685 ± 37.4	668 ± 39.4	484 ± 16.1	4729 ± 8.6	7 ± 0.4	480 ± 5.7	78 ± 0.2	23 ± 0.1	564 ± 6.0

**Table 9:** Pearson’s correlation coefficients (*r*) of individual lipid content with in vitro starch digestion of rice samples.

Rice Lipids	Correlation (*r*) between Rice Lipids and In Vitro Starch Digestibility									
	R1, R2, R4–R10 (*n* = 9) ^a^					R11–R25 (*n* = 15) ^b^				
	Glucose Concentration at:			AUC at:		Glucose Concentration at:			AUC at:	
	60 min	120 min	180 min	0–120 min	0–180 min	60 min	120 min	180 min	0–120 min	0–180 min
TAG 50:2	−0.42	−0.30	−0.29	−0.38	−0.38	−0.01	−0.38	−0.51	−0.18	−0.37
TAG 52:2	−0.50	−0.23	−0.04	−0.41	−0.32	−0.02	−0.32	−0.50	−0.17	−0.35
TAG 52:3	−0.51	−0.21	0.01	−0.40	−0.30	−0.06	−0.33	−0.52 *	−0.21	−0.38
TAG 52:4	−0.54	−0.39	−0.35	−0.49	−0.49	−0.19	−0.34	−0.61 *	−0.32	−0.48
TAG 54:1	−0.42	−0.09	0.15	−0.30	−0.18	−0.16	−0.54 *	−0.61 *	−0.39	−0.57 *
TAG 54:3	−0.44	−0.08	0.19	−0.31	−0.17	−0.08	−0.46	−0.63 *	−0.29	−0.49
TAG 54:4	−0.44	−0.07	0.24	−0.30	−0.15	−0.18	−0.47	−0.69 *	−0.37	−0.57 *
TAG 54:6	−0.51	−0.18	0.11	−0.39	−0.27	−0.25	−0.42	−0.68 *	−0.40	−0.56 *
Total TAG	−0.52	−0.19	0.05	−0.40	−0.29	−0.11	−0.39	−0.61 *	−0.28	−0.47
DAG 34:2	0.02	0.22	0.15	0.10	0.16	−0.35	−0.44	−0.69 *	−0.42	−0.58 *
DAG 36:4	−0.08	0.16	0.23	0.02	0.10	−0.38	−0.47	−0.76 *	−0.53 *	−0.68 *
DAG 36:3	−0.05	0.28	0.35	0.08	0.20	−0.35	−0.48	−0.77 *	−0.51	−0.67 *
DAG 36:2	0.02	0.35	0.35	0.16	0.27	−0.32	−0.48	−0.76 *	−0.49	−0.66 *
Total DAG	−0.03	0.27	0.30	0.09	0.20	−0.33	−0.47	−0.76 *	−0.50	−0.66 *
PC 36:4	−0.49	−0.28	0.10	−0.42	−0.32	0.28	−0.07	0.17	0.19	0.14
PC 36:2	−0.42	−0.22	0.17	−0.35	−0.24	0.06	−0.24	0.04	−0.06	−0.09
PC 34:1	−0.42	−0.25	0.13	−0.36	−0.26	0.11	−0.14	0.03	0.02	−0.02
PC 34:2	−0.46	−0.29	0.08	−0.41	−0.32	0.35	−0.02	0.16	0.28	0.20
Total PC	−0.45	−0.26	0.13	−0.39	−0.28	0.22	−0.13	0.11	0.12	0.07
LPC18:3	−0.42	−0.46	0.01	−0.45	−0.40	0.28	−0.04	0.04	0.21	0.12
LPC18:2	−0.46	−0.62	−0.29	−0.54	−0.57	−0.08	−0.48	−0.53 *	−0.30	−0.47
LPC18:1	−0.26	−0.09	0.31	−0.20	−0.08	−0.52	−0.31	−0.44	−0.57 *	−0.55 *
LPC14:0	−0.24	−0.57	−0.60	−0.39	−0.53	0.40	0.13	0.48	0.39	0.41
LPC16:0	−0.47	−0.62	−0.49	−0.55	−0.62	−0.27	0.00	−0.42	−0.22	−0.26
Total LPC	−0.51	−0.68 *	−0.42	−0.60	−0.65	−0.18	−0.21	−0.41	−0.25	−0.33
LPE18:3	−0.63	−0.54	−0.07	−0.61	−0.54	0.10	−0.08	−0.11	0.04	−0.03
LPE18:2	−0.61	−0.64	−0.36	−0.64	−0.65	−0.27	−0.20	−0.53 *	−0.31	−0.41
LPE18:1	−0.29	−0.12	0.17	−0.23	−0.14	−0.54 *	−0.29	−0.44	−0.58 *	−0.55 *
LPE14:0	−0.41	−0.66	−0.71 *	−0.53	−0.66	0.21	0.01	0.40	0.17	0.23
LPE16:0	−0.49	−0.56	−0.40	−0.54	−0.57	−0.48	−0.32	−0.46	−0.55 *	−0.55 *
Total LPE	−0.63	−0.66	−0.39	−0.66	−0.68 *	−0.42	−0.29	−0.48	−0.49	−0.51
Total LPL	−0.53	−0.69 *	−0.42	−0.62	−0.66	−0.22	−0.23	−0.44	−0.29	−0.37

* Correlations are significant at *p* < 0.05; ^a,b^ the rice samples were analyzed separately to avoid the effect from AAC variation. ^a^ Purchased commercial rice samples (R1, R2, R4–R10); R3 is a glutinous rice and was excluded due to the large effect of low AAC. ^b^ The rice samples acquired from Bangladesh (R11–R25) with relatively similar AAC.

**Table 10:** In vitro rice starch digestibility upon addition of lipids to and removal of lipids from rice flour (Doongara, R02).

Treatment	AUC at	
	0–120 min	0–180 min
R − L	68.9 ± 1.12 ^e^	136.8 ± 1.17 ^d^
R + L	58.9 ± 0.23 ^a^	121.0 ± 0.92 ^a^
R + TAG	61.1 ± 1.45 ^abc^	123.9 ± 1.87 ^ab^
R + DAG	62.3 ± 0.64 ^bcd^	128.3 ± 1.11 ^bc^
R + PC	63.7 ± 0.40 ^cd^	128.2 ± 1.77 ^c^
R + LPL	60.0 ± 1.29 ^ab^	122.5 ± 2.74 ^a^
R+ WSB	64.5 ± 1.74 ^d^	131.3 ± 3.04 ^c^
R + PPL	64.5 ± 0.75 ^d^	131.5 ± 0.93 ^c^
R	64.0 ± 1.31 ^d^	131.9 ± 1.43 ^c^

Data are represented as mean ± standard deviations (*n* = 3); columns with same superscript letters are not significantly different according to Duncan’s multiple range test (*p* < 0.05); R − L: lipid removed from rice flour; R + L: WSB-extracted lipid added to rice flour; R + TAG: TAG 54:6 added to rice flour; R + DAG: DAG 36:4 added to rice flour; R + PC: PC 36:2 added to rice flour; R + LPC: LPC 16:0 added to rice flour; R + WSB: water-saturated-butanol-treated control rice flour; R + PPL: 75% *n*-propanol-treated control rice flour; R: untreated control rice flour; AUC: area under curve for digestion period (minute) vs. mg glucose released/100 mg dry rice flour.

**Table 11:** Pearson’s correlation coefficients (*r*) between lipid-bound amylose content and apparent amylose content in defatted rice flour with in vitro starch digestion of rice samples (*n* = 25).

Rice Group	Correlation (*r*) between AAC and In Vitro Starch Digestibility				
	Glucose Concentration at:			AUC at:	
	60 min	120 min	180 min	0–120 min	0–180 min
AAC-L *	−0.23	0.17	−0.10	−0.08	−0.02
Lipid-bound amylose	−0.02	0.24	−0.02	0.09	0.12

* AAC-L: apparent amylose content in defatted rice samples.

## Data Availability

The data is contained within the article.

## References

[1] Kaur B., Ranawana V., Henry J. (2016). The Glycemic Index of Rice and Rice Products: A Review, and Table of GI Values. Crit. Rev. Food Sci. Nutr..

[2] Khatun A., Waters D.L.E., Liu L. (2019). A Review of Rice Starch Digestibility: Effect of Composition and Heat-Moisture Processing. Starch-Stärke.

[3] Dhital S., Butardo V.M., Jobling S.A., Gidley M.J. (2015). Rice starch granule amylolysis—Differentiating effects of particle size, morphology, thermal properties and crystalline polymorph. Carbohydr. Polym..

[4] Syahariza Z.A., Sar S., Hasjim J., Tizzotti M.J., Gilbert R.G. (2013). The importance of amylose and amylopectin fine structures for starch digestibility in cooked rice grains. Food Chem..

[5] Sujatha S.J., Ahmad R., Bhat P.R. (2004). Physicochemical properties and cooking qualities of two varieties of raw and parboiled rice cultivated in the coastal region of Dakshina Kannada, India. Food Chem..

[6] Kaur K., Singh N. (2000). Amylose-lipid complex formation during cooking of rice flour. Food Chem..

[7] Choudhury N.H., Juliano B.O. (1980). Lipids in developing and mature rice grain. Phytochemistry.

[8] Yu S., Ma Y., Sun D.-W. (2009). Impact of amylose content on starch retrogradation and texture of cooked milled rice during storage. J. Cereal Sci..

[9] Guraya H.S., Kadan R.S., Champagne E.T. (1997). Effect of Rice Starch-Lipid Complexes on In Vitro Digestibility, Complexing Index, and Viscosity. Cereal Chem..

[10] Kaur B., Ranawana V., Teh A., Henry C.J.K. (2015). The Glycemic Potential of White and Red Rice Affected by Oil Type and Time of Addition. J. Food Sci..

[11] Farooq A.M., Dhital S., Li C., Zhang B., Huang Q. (2018). Effects of palm oil on structural and in vitro digestion properties of cooked rice starches. Int. J. Biol. Macromol..

[12] Chen X., He X., Fu X., Zhang B., Huang Q. (2017). Complexation of rice starch/flour and maize oil through heat moisture treatment: Structural, in vitro digestion and physicochemical properties. Int. J. Biol. Macromol..

[13] Seneviratne H.D., Biliaderis C.G. (1991). Action of α-amylases on amylose-lipid complex superstructures. J. Cereal Sci..

[14] Biliaderis C.G., Galloway G. (1989). Crystallization behavior of amylose-V complexes: Structure-property relationships. Carbohydr. Res..

[15] Chung O.K., Ohm J.B., Kulp K., Ponte J.G.J. (2000). Cereal lipids. Handbook of Cereal Science and Technology.

[16] Morrison W.R. (1981). Starch lipids: A reappraisal. Starch.

[17] Azudin M.N., Morrison W.R. (1986). Non-starch lipids and starch lipids in milled rice. J. Cereal Sci..

[18] Chung O.K., Ohm J.B., Ram M.S., Park S., Howitt C.A., Khan K., Shewry P.R. (2009). Wheat lipids. Wheat Chemistry and Technology.

[19] Juliano B.O. (1983). Lipids in Rice and Rice Processing. Lipids in Cereal Technology.

[20] Hu P., Fan X., Lin L., Wang J., Zhang L., Wei C. (2018). Effects of surface proteins and lipids on molecular structure, thermal properties, and enzymatic hydrolysis of rice starch. Food Sci. Technol..

[21] Ye J., Hu X., Luo S., McClements D.J., Liang L., Liu C. (2018). Effect of endogenous proteins and lipids on starch digestibility in rice flour. Food Res. Int..

[22] Liu L., Tong C., Bao J., Waters D.L.E., Rose T.J., King G.J. (2014). Determination of Starch Lysophospholipids in Rice Using Liquid Chromatography–Mass Spectrometry (LC-MS). J. Agric. Food Chem..

[23] Blazek J. (2009). Role of Amylose in Structure-Function Relationship in Starches from Australian Wheat Varieties.

[24] Chrastil J. (1987). Improved colorimetric determination of amylose in starches or flours. Carbohydr. Res..

[25] Tong C., Chen Y., Tang F., Xu F., Huang Y., Chen H., Bao J. (2014). Genetic diversity of amylose content and RVA pasting parameters in 20 rice accessions grown in Hainan, China. Food Chem..

[26] Geng P., Harnly J.M., Chen P. (2015). Differentiation of Whole Grain from Refined Wheat (*T. aestivum*) Flour Using Lipid Profile of Wheat Bran, Germ, and Endosperm with UHPLC-HRAM Mass Spectrometry. J. Agric. Food Chem..

[27] Liu L., Guo Q., He Z., Xia X., Waters D.L.E., Raymond C.A., King G.J. (2017). Genotypic Variation in Wheat Flour Lysophospholipids. Molecules.

[28] Zhou Z., Robards K., Helliwell S., Blanchard C. (2007). Effect of the addition of fatty acids on rice starch properties. Food Res. Int..

[29] Khatun A., Waters D.L.E., Liu L. (2018). Optimization of an in vitro starch digestibility assay for rice. Starch-Stärke.

[30] Khatun A., Waters D.L.E., Liu L. (2020). The impact of rice protein on in vitro rice starch digestibility. Food Hydrocoll..

[31] Goddard M.S., Young G., Marcus R. (1984). The effect of amylose content on insulin and glucose responses to ingested rice. Am. J. Clin. Nutr..

[32] De Guzman M.K., Parween S., Butardo V.M., Alhambra C.M., Anacleto R., Seiler C., Bird A.R., Chow C.-P., Sreenivasulu N. (2017). Investigating glycemic potential of rice by unraveling compositional variations in mature grain and starch mobilization patterns during seed germination. Sci. Rep..

[33] Chung H.-J., Liu Q., Lee L., Wei D. (2011). Relationship between the structure, physicochemical properties and in vitro digestibility of rice starches with different amylose contents. Food Hydrocoll..

[34] Zhang W., Bi J., Yan X., Wang H., Zhu C., Wang J., Wan J. (2007). In vitro measurement of resistant starch of cooked milled rice and physico-chemical characteristics affecting its formation. Food Chem..

[35] Howlader M.Z.H., Biswas S.K. (2009). Screening for nutritionally rich and low glycemic index Bangladeshi rice varieties. Final Rep. CF.

[36] Panlasigui L.N., Thompson L.U., Juliano B.O., Pérez C.M., Yiu S.H., Greenberg G.R. (1991). Rice varieties with similar amylose content differ in starch digestibility and glycemic response in humans. Am. J. Clin. Nutr..

[37] Pang Y.L., Ali J., Wang X.Q., Franje N.J., Revilleza J.E., Xu J.L., Li Z.K. (2016). Relationship of Rice Grain Amylose, Gelatinization Temperature and Pasting Properties for Breeding Better Eating and Cooking Quality of Rice Varieties. PLoS ONE.

[38] Raphaelides S., Karkalas J. (1988). Thermal dissociation of amylose-fatty acid complexes. Carbohydr. Res..

[39] Fujino Y., Miyazawa T. (1976). Neutral Lipids Present in Starch of Uruchi and Mochi Rice. Starch-Stärke.

[40] Mano Y., Kawaminami K., Kojima M., Ohnishi M. (1999). Comparative Composition of Brown Rice Lipids (Lipid Fractions) of Indica and Japonica Rices. Biosci. Biotechnol. Biochem..

[41] Christie W.W. (1993). Preparation of ester derivatives of fatty acids for chromatographic analysis. Adv. Lipid Methodol..

[42] Ishikawa T., Ito Y., Kawai-Yamada M. (2016). Molecular characterization and targeted quantitative profiling of the sphingolipidome in rice. Plant J..

[43] Zeb A., Murkovic M. (2010). Analysis of triacylglycerols in refined edible oils by isocratic HPLC-ESI-MS. Eur. J. Lipid Sci. Technol..

[44] Bahrami N., Yonekura L., Linforth R., Da Silva M.C., Hill S., Penson S., Chope G., Fisk I.D. (2014). Comparison of ambient solvent extraction methods for the analysis of fatty acids in non-starch lipids of flour and starch. J. Sci. Food Agric..

[45] Tong C., Liu L., Waters D.L.E., Rose T.J., Bao J., King G.J. (2014). Genotypic Variation in Lysophospholipids of Milled Rice. J. Agric. Food Chem..

[46] Choudhury N.H., Juliano B.O. (1980). Effect of amylose content on the lipids of mature rice grain. Phytochemistry.

[47] Eliasson A.-C., Krog N. (1985). Physical properties of amylose-monoglyceride complexes. J. Cereal Sci..

[48] Chao C., Yu J., Wang S., Copeland L., Wang S. (2018). Mechanisms Underlying the Formation of Complexes between Maize Starch and Lipids. J. Agric. Food Chem..

[49] Rattanamechaiskul C., Soponronnarit S., Prachayawarakorn S. (2014). Glycemic response to brown rice treated by different drying media. J. Food Eng..

[50] Luo J., Liu L., Konik-Rose C., Tian L., Singh S., Howitt C.A., Li Z., Liu Q. (2021). Down-Regulation of *FAD2-1* Gene Expression Alters Lysophospholipid Composition in the Endosperm of Rice Grain and Influences Starch Properties. Foods.

[51] Debet M.R., Gidley M.J. (2006). Three classes of starch granule swelling: Influence of surface proteins and lipids. Carbohydr. Polym..

[52] Tong C., Liu L., Waters D.L.E., Huang Y., Bao J. (2015). The contribution of lysophospholipids to pasting and thermal properties of nonwaxy rice starch. Carbohydr. Polym..

[53] Derycke V., Vandeputte G.E., Vermeylen R., De Man W., Goderis B., Koch M.H.J., Delcour J.A. (2005). Starch gelatinization and amylose–lipid interactions during rice parboiling investigated by temperature resolved wide angle X-ray scattering and differential scanning calorimetry. J. Cereal Sci..

[54] Tufvesson F., Wahlgren M., Eliasson A.-C. (2003). Formation of Amylose-Lipid Complexes and Effects of Temperature Treatment. Part 1. Monoglycerides. Starch-Stärke.

